# Deficiency of TMEM16F in hair cells prevents diabetes-related and noise-induced hearing loss

**DOI:** 10.1016/j.gendis.2025.101708

**Published:** 2025-06-06

**Authors:** Liyuan Chen, Na Wang, Jiajia Li, Yan Li, Li Yi, Yupeng Ling, Qiuling Zhao, Yibing Fang, Guilan Chen, Bo Zeng

**Affiliations:** a Key Laboratory of Medical Electrophysiology, Ministry of Education & Medical Electrophysiological Key Laboratory of Sichuan Province, Institute of Cardiovascular Research, Southwest Medical University, Luzhou, Sichuan 646000, China; b Institute of Pathology and Southwest Cancer Center, Southwest Hospital, Third Military Medical University (Army Medical University), and Key Laboratory of Tumor Immunopathology, Ministry of Education of China, Chongqing 400038, China; c Cardiac Surgery, Southwest Hospital, Third Military Medical University (Army Medical University), Chongqing 400038, China; d School of Life and Health Sciences, Hainan Province Key Laboratory of One Health, Collaborative Innovation Center of Life and Health, Hainan University, Haikou, Hainan 570228, China

Hearing loss is a common health condition associated with genetic variations, diseases, loud noise exposure, and aging. Diabetes mellitus, one of the most prevalent chronic diseases, is known to increase the incidence of hearing loss in humans. However, no effective therapeutics have been developed to treat diabetes-related hearing loss (DRHL). The most evident histopathologic characteristic in the cochleae of diabetic patients is degeneration of the stria vascularis and cochlear outer hair cells (OHCs).^1^ For noise-induced hearing loss (NIHL), damage to hair cells is also a principal cause.^2^ Therefore, promoting hair cell survival would be critical for preventing DRHL and NIHL.

Transmembrane protein 16F (TMEM16F) is a Ca^2+^-activated non-selective ion channel and phospholipid scramblase that disrupts the asymmetric distribution of different phospholipids between the inner and outer leaflets of the plasma membrane, causing exposure of phosphatidylserine on the cell surface. TMEM16F has been found to participate in multiple cell–death pathways such as phagocytosis, necroptosis, ferroptosis, and pyroptosis.^3^ Although the mechanisms of DRHL and NIHL are not well understood, oxidative stress and dysregulated Ca^2+^ signaling in the cochlea have been generally proposed.^4^ Cellular Ca^2+^ overload is commonly observed in many tissues under diabetic conditions, and loud noise also induces Ca^2+^ overload in hair cells by stimulating the mechanoelectrical transduction channel and other pathways.^2^ Since TMEM16F is activated by elevated intracellular Ca^2+^, it may contribute to cell death under these conditions. We hypothesized that a deficiency of TMEM16F activity would be beneficial for cochlear cell survival and tested its effect on hearing using TMEM16F knockout (KO) mice.

With CAG promoter-driven ubiquitous expression of Cre recombinase (CAG-CreERT), TMEM16F KO in all tissues and organs was induced by tamoxifen injection in adult mice. Depletion of TMEM16F in cochleae was confirmed by western blotting (Fig. 1A). Type 1 diabetes was induced by streptozotocin injection and the auditory brainstem response (ABR) test with click stimuli showed that TMEM16F KO mice were much less susceptible to DRHL in 3 months (from 3 to 6 months of age), with ABR thresholds of 30–60 dB for KO mice and 60–90 dB for control (FF) mice in the 3rd month (Fig. 1B, C). The ABR thresholds to fixed-frequency tones were also much lower in KO mice (Fig. S1A). The function of OHCs, as indicated by distortion product otoacoustic emission (DPOAE), was severely impaired in FF mice but significantly less affected in KO mice (Fig. 1D). FF and KO mice without streptozotocin injection (non-diabetic control) both exhibited progressive hearing impairment but did not reach a significant level in the same 3-month period (Fig. 1B–D). No difference was observed between male and female mice in the hearing test, and the body weights were comparable between FF and KO mice at the end of the experiment (Fig. S2).

**Figure 1:**
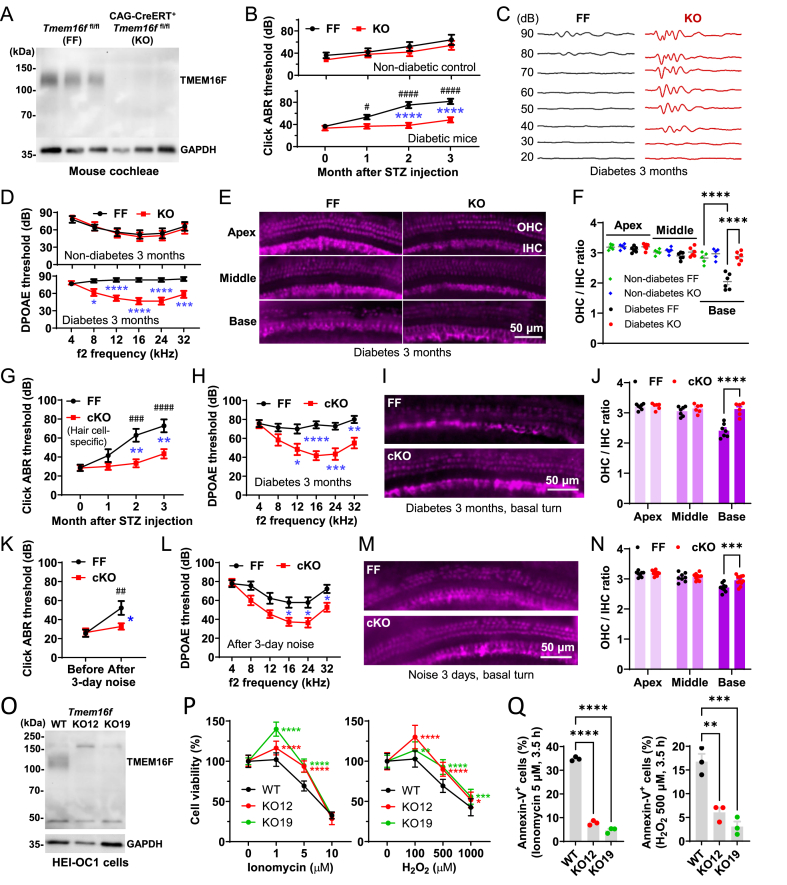
TMEM16F knockout prevented hearing loss and hair cell death in mice. **(A)** Western blotting confirmed the depletion of TMEM16F in mouse cochleae with Cre recombinase. **(B)** Auditory brainstem response (ABR) thresholds evoked by click stimuli in control (FF) and knockout (KO) mice during 3 months of non-diabetic or diabetic conditions. *n* = 5 (non-diabetic) or 6 (diabetic) mice in each group. **(C)** Click-evoked ABR waveforms at the end of the 3 months. **(D)** Distortion product otoacoustic emission (DPOAE) thresholds of the mice at month 3. *n* = 5 or 6 mice in each group. **(E)** Immunofluorescence (myosin-7a) of hair cells in mouse cochleae. **(F)** The ratio of the numbers of outer and inner hair cells. *n* = 5 or 6 mice in each group. **(G, H)** Click-evoked ABR thresholds in hair cell-specific cKO and FF mice during 3 months of diabetic condition, and DPOAE thresholds at month 3. *n* = 7 (FF) and 6 (cKO) mice. **(I, J)** Immunofluorescence and the ratio of the outer and inner hair cells. *n* = 7 (FF) and 6 (cKO) mice. **(K, L)** Click-evoked ABR thresholds in FF and cKO mice treated with 120-dB noise, and DPOAE thresholds after the treatment. *n* = 9 (FF) and 11 (cKO) mice. **(M, N)** Immunofluorescence and the ratio of the outer and inner hair cells. *n* = 9 (FF) and 11 (cKO) mice. **(O)** Western blotting confirmed TMEM16F KO in two lines of HEI-OC1 cells (immortalized mouse hair cells). **(P)** Viability of wild-type (WT) and TMEM16F–KO HEI-OC1 cells treated with ionomycin for 12 h (*n* = 14 wells) or H_2_O_2_ for 6 h (*n* = 16 wells) assessed by WST-1 assay. **(Q)** Percentage of cells with phosphatidylserine exposure detected by flow cytometry after treatment with 5 μM ionomycin or 500 μM H_2_O_2_ for 3.5 h. *n* = 3 dishes of cells in each group. ^#^*P* < 0.05, ^##^*P* < 0.01, ^###^*P* < 0.001, and ^####^*P* < 0.0001 versus month 0 (B and G) or before (K), by 2-way ANOVA and Sidak's test; ∗*P* < 0.05, ∗∗*P* < 0.01, ∗∗∗*P* < 0.001, and ∗∗∗∗*P* < 0.0001 between FF and KO (B, D and F) or cKO (G, H, J, K, L and N), or between WT and KO (P and Q), by 2-way ANOVA and Sidak's test (B, D, F, G, H, J, K, L, N, and P) or 1-way ANOVA and Tukey's test (Q).

Immunofluorescence of hair cells in whole-mount cochleae revealed massive OHC loss in the basal turn in diabetic FF mice, while most OHCs in KO mice survived the 3-month diabetic period (Fig. 1E). In both groups, the inner hair cells were largely unaffected, so they were used to normalize the numbers of OHCs (Fig. 1E, F).

To confirm whether the improvement of hearing by TMEM16F KO is mainly due to the protection of OHCs, we generated hair cell-specific TMEM16F conditional KO (cKO) mice with Pou4f3-CreERT2. Similar to the global KO mice, the cKO mice were more resistant to hearing loss through 3 months of diabetic condition. The ABR and DPOAE thresholds of the cKO mice were significantly lower than those control littermates (Fig. 1G, H; Fig. S1B). Severe OHC loss in the basal turn was observed in the control mice, which was negligible in the cKO mice (Fig. 1I, J).

We also used 120-dB intense noise to induce hearing impairment in the mice. After 3-day noise treatment (3.5 h/day), the ABR and DPOAE thresholds of the cKO mice remained low, while those of the control mice were dramatically increased (Fig. 1K, L; Fig. S1C). The OHC loss in the cKO mice was markedly less than the control mice (Fig. 1M, N).

In both diabetic and noise-treated FF mice, we found that cleaved caspase-3, a marker of apoptosis, was abundant in regions with massive OHC loss, while cochleae from the cKO mice were largely negative for this marker (Fig. S3A). On the other hand, phosphorylated mixed lineage kinase domain-like protein (MLKL), a marker of necrosis, was not detected in all samples (Fig. S3B). These results suggest that apoptosis may be the major cause of hair cell death in FF mice, and TMEM16F is actively involved in this process.

We then employed an immortalized hair cell line, HEI-OC1, to confirm the effect of TMEM16F deficiency on hair cell viability under stress conditions. Since HEI-OC1 cells essentially require high glucose in culture medium for growth, the conventional high/low-glucose culture method cannot be used to mimic the diabetic condition. For both DRHL and NIHL, Ca^2+^ overload and oxidative stress are critical players in hair cell injuries.^4^ Therefore, ionomycin and H_2_O_2_ were used to induce Ca^2+^ overload and oxidative stress in HEI-OC1 cells, respectively. We generated two TMEM16F–KO cell lines (Fig. 1O) and found that the KO cells proliferated faster than wild-type cells under low concentrations of ionomycin (1 μM) or H_2_O_2_ (100 μM), while the viability of both KO cell lines after treatment with high concentrations of ionomycin (5 and 10 μM) or H_2_O_2_ (500 and 1000 μM) was significantly higher than wild-type cells (Fig. 1P). These results suggest that TMEM16F plays a negative role in hair cell survival. The percentage of cells with surface phosphatidylserine exposure, as revealed by annexin-V flow cytometry, was considerably less in TMEM16F–KO cells than in wild-type cells treated with 5 μM ionomycin or 500 μM H_2_O_2_ (Fig. 1Q; Fig. S4), confirming that the KO cells lack TMEM16F activity.

In summary, we identified TMEM16F as a promising drug target for the prevention of DRHL and NIHL. Promoting hair cell survival by TMEM16F inhibition may be a widely applicable strategy for intervention of more types of acquired hearing impairment.

## CRediT authorship contribution statement

**Liyuan Chen:** Methodology, Investigation. **Na Wang:** Writing – original draft, Methodology, Investigation, Funding acquisition. **Jiajia Li:** Investigation. **Yan Li:** Investigation. **Li Yi:** Investigation. **Yupeng Ling:** Investigation. **Qiuling Zhao:** Investigation. **Yibing Fang:** Investigation. **Guilan Chen:** Writing – review & editing, Supervision, Funding acquisition, Conceptualization. **Bo Zeng:** Writing – review & editing, Writing – original draft, Supervision, Funding acquisition, Conceptualization.

## Ethics declaration

All the animal experimental procedures were approved by the Ethical Committee of Southwest Medical University and were in accordance with the national guidelines for the care and use of laboratory animals.

## Data availability

Data obtained in this study are available from the corresponding authors upon reasonable request.

## Funding

This study was supported by the 10.13039/501100001809National Natural Science Foundation of China (No. 32171106), the Sichuan Science and Technology Program (China) (No. 2022YFS0607, 2022YFS0627), the Joint Research Program of Luzhou City and Southwest Medical University (Sichuan, China) (No. 2020LZXNYDJ21), and the Science and Technology Program of Luzhou City, Sichuan, China (No. 2024JYJ043).

## Conflict of interests

The authors declared no competing interests.
