# Supplementary material for: Deficiency of TMEM16F in hair cells prevents diabetes-related and noise-induced hearing loss
Source: Genes Dis. 2025 Jun 6;13(2):101708. doi: 10.1016/j.gendis.2025.101708 (PMC12648708; doi:10.1016/j.gendis.2025.101708)
Supplement: Multimedia component 1 [file mmc1.docx]

**Vulnerability of hair cells in the basal turn of cochlea**

Vulnerability of the basal turn is a common feature in almost all types of cochlear damage [1]. The basal turn encodes high-frequency sounds and thus suffers from more frequent mechanical stress [1]. Hair cells in the basal turn have higher metabolic demand and sensitivity to free radicals, which make it the most susceptible region of the cochlea to damage [2, 3]. Compared to the basal turn, the apical and middle turns are more resistant to damage, so there were very few hair cell loss in these regions in both FF and KO mice (Figure 1E and F, comparing with non-diabetic mice).

**Involvement of TMEM16F activities in apoptosis**

Phospholipid scrambling and non-selective ion permeation are two known functions of TMEM16F related to apoptosis. As scramblase-induced PS exposure is a downstream event in apoptosis that occurs after caspase-3 activation [4], deficiency of PS exposure might not prevent caspase-3 cleavage. However, non-selective ion permeation through the plasma membrane can induce apoptosis by disrupting cellular ion homeostasis (e.g., overload of Ca^2+^), leading to mitochondrial dysfunction and activation of apoptotic pathways [5]. Therefore, when TMEM16F is activated by increased Ca^2+^ in hair cells under oxidative stress or noise stimulation, Ca^2+^ influx through the channel pore could exacerbate Ca^2+^ overload and cause excessive Ca²⁺ uptake by mitochondria, leading to mitochondrial membrane permeabilization (MMP) and the opening of the mitochondrial permeability transition pore (mPTP). This results in the release of pro-apoptotic factors such as cytochrome c, which activates caspases and initiates apoptosis [6]. In the absence of TMEM16F-mediated Ca^2+^ influx, intracellular apoptotic signals may be limited below the threshold to trigger mitochondrial dysfunction and caspase activation. Deficiency of TMEM16F-mediated lipid scrambling may further prevent or delay PS exposure and apoptosis.

**The role of TMEM16A in the cochlea**

Previous studies have revealed the critical role of TMEM16A, a Ca^2+^-activated Cl^-^ channel of the TMEM16 family, in the development of cochlea. TMEM16A is detected in basal cells of the stria vascularis, and efferent nerve endings and supporting cells under hair cells, but not in hair cells [7, 8]. In supporting cells, TMEM16A-mediated Cl^-^ secretion drives K^+^ release, which induces periodic excitation of hair cells and spiral ganglion neurons in the developing cochlea [9]. TMEM16A also controls the maturation of spiral ganglion neurons by modulating their membrane excitability [10]. By regulating the spontaneous activity of hair cells and spiral ganglion neurons in cochlea, TMEM16A can further impact the development of auditory circuits in the brainstem [11, 12]. Although the role of TMEM16A in cochlear development has been well recognized, little is known about its function in mature cochlea. Application of a TMEM16A inhibitor, T16Ainh-A01, significantly increased the ABR threshold in 3-month-old guinea pigs [13], suggesting that TMEM16A inhibition may be harmful to auditory function. However, the specificity of the drug should always be taken into consideration, and inducible TMEM16A KO in adult mice is required to confirm this conclusion.

**References**

1. Fettiplace R, and Nam JH. Tonotopy in calcium homeostasis and vulnerability of cochlear hair cells. Hear Res*.* 2019;376:11-21.

2. Sha SH, Taylor R, Forge A, and Schacht J. Differential vulnerability of basal and apical hair cells is based on intrinsic susceptibility to free radicals. Hear Res*.* 2001;155(1-2):1-8.

3. Pouyatos B, Gearhart CA, Nelson-Miller A, Fulton S, and Fechter LD. Selective vulnerability of the cochlear Basal turn to acrylonitrile and noise. J Toxicol*.* 2009;2009:908596.

4. Sakuragi T, and Nagata S. Regulation of phospholipid distribution in the lipid bilayer by flippases and scramblases. Nat Rev Mol Cell Biol*.* 2023;24(8):576-96.

5. Orrenius S, Zhivotovsky B, and Nicotera P. Regulation of cell death: the calcium-apoptosis link. Nat Rev Mol Cell Biol*.* 2003;4(7):552-65.

6. Mustafa M, Ahmad R, Tantry IQ, Ahmad W, Siddiqui S, Alam M, et al. Apoptosis: A Comprehensive Overview of Signaling Pathways, Morphological Changes, and Physiological Significance and Therapeutic Implications. Cells*.* 2024;13(22).

7. Jeon JH, Park JW, Lee JW, Jeong SW, Yeo SW, and Kim IB. Expression and immunohistochemical localization of TMEM16A/anoctamin 1, a calcium-activated chloride channel in the mouse cochlea. Cell Tissue Res*.* 2011;345(2):223-30.

8. Yi E, Lee J, and Lee CJ. Developmental Role of Anoctamin-1/TMEM16A in Ca(2+)-Dependent Volume Change in Supporting Cells of the Mouse Cochlea. Exp Neurobiol*.* 2013;22(4):322-9.

9. Wang HC, Lin CC, Chong R, Zhang-Hooks Y, Agarwal A, Ellis-Davies G, et al. Spontaneous Activity of Cochlear Hair Cells Triggered by Fluid Secretion Mechanism in Adjacent Support Cells. Cell*.* 2015;163(6):1348-59.

10. Zhang XD, Lee JH, Lv P, Chen WC, Kim HJ, Wei D, et al. Etiology of distinct membrane excitability in pre- and posthearing auditory neurons relies on activity of Cl- channel TMEM16A. Proc Natl Acad Sci U S A*.* 2015;112(8):2575-80.

11. Maul A, Huebner AK, Strenzke N, Moser T, Rübsamen R, Jovanovic S, et al. The Cl(-)-channel TMEM16A is involved in the generation of cochlear Ca(2+) waves and promotes the refinement of auditory brainstem networks in mice. Elife*.* 2022;11.

12. Kersbergen CJ, Babola TA, Rock J, and Bergles DE. Developmental spontaneous activity promotes formation of sensory domains, frequency tuning and proper gain in central auditory circuits. Cell Rep*.* 2022;41(7):111649.

13. Zhou Y, Song J, Wang YP, Zhang AM, Tan CY, Liu YH, et al. Age‑associated variation in the expression and function of TMEM16A calcium‑activated chloride channels in the cochlear stria vascularis of guinea pigs. Mol Med Rep*.* 2019;20(2):1593-604.
