# Supplementary material for: Deficiency of TMEM16F in hair cells prevents diabetes-related and noise-induced hearing loss
Source: Genes Dis. 2025 Jun 6;13(2):101708. doi: 10.1016/j.gendis.2025.101708 (PMC12648708; doi:10.1016/j.gendis.2025.101708)
Supplement: Multimedia component 2 [file mmc2.docx]

**Methods**

***Mice***

CAG-CreERT and *Tmem16f* ^fl/+^ mice of C57BL/6 background were purchased from Cyagen (Suzhou, China). Pou4f3-CreERT2 mice were gifts from Dr. Guoqiang Wan at Nanjing University, China. KO and cKO mice were bred in the Experimental Animal Center of Southwest Medical University in specific pathogen free (SPF) conditions with standard 12-h light and dark cycles and free access to food and water.

***ABR and DPOAE tests***

ABR and DPOAE recordings in isoflurane-anesthetized mice were performed using the RZ6 system with BioSigRZ software (Tucker-Davis Technologies, USA). For ABR test, click and pure tone stimuli at frequencies from 6 to 30 kHz were applied and the sound pressure level (SPL) was from 10 to 90 dB by 10-dB step. ABR threshold was defined as the lowest sound intensity that clearly evoked the first wave. DPOAEs in response to primary tones f1 and f2 were recorded at 2×f1–f2, where the f2/f1 ratio was set to 1.2, and the f2 level is 10 dB lower than the f1 level. DPOAE threshold was calculated as the f2 level required to produce a response at 5 dB SPL.

***Diabetic model***

KO, cKO and FF mice received intraperitoneal injection of tamoxifen (75 mg/kg per day) for 5 consecutive days at the age of 8 weeks. Two weeks after tamoxifen injection, the mice were intraperitoneally injected with STZ in citrate buffer (55 mg/kg body weight, pH 4.5) once a day for 5 days. Blood glucose was measured after a week and mice with blood glucose over 16.7 mM were used in further studies.

***Noise treatment***

Awake mice were placed in a soundproof chamber and received broadband noise of 120 dB for 3 days (3.5 h/day), which is an optimized condition to cause hearing impairment but not animal death. Auditory tests were performed before and after the 3-days noise treatment.

***Immunofluorescence***

The temporal bones were fixed with 4% paraformaldehyde at room temperature (RT). After fixation, paraformaldehyde was removed and replaced with 120 mM ethylenediaminetetraacetic acid (EDTA) to decalcify the temporal bone for 2 days at RT on a 3D rotator. Fresh EDTA solution was changed every 12 h. The organ of Corti was dissected from the cochlea. The apex, middle turn and basal turn were permeabilized and blocked with 1% Triton X-100, 1% bovine serum albumin, and 10% normal horse serum diluted in 10 mM PBS (pH 7.4) for 1 h at RT on a 3D rotator. The samples were incubated with the primary antibody (1:100 dilution of rabbit anti-myosin-7a (Proteus 25-6790), Cleaved Caspase-3 (CST 9661S), or Phosphorylated MLKL (Abiowell AWA10442)) overnight, and then the secondary antibody (AF647 Donkey anti-Rabbit IgG, Invitrogen A31573, 1:200) for 2 h at RT. At last, the samples were mounted and imaged on an Eclipse Ti-E inverted microscope (Nikon, Japan). OHC and IHC in the same region are counted, and the ratio between the total number of OHC and the total number of IHC in the same turn of a same cochlea is calculated.

***TMEM16F KO in HEI-OC1 cells***

HEI-OC1 cells were purchased from OriCell (Guangzhou, China) and cultured in DMEM with 10% fetal bovine serum (FBS), 100 units/ml penicillin and 100 μg/ml streptomycin in a 5% CO_2_ incubator at 37°C. The cells were transfected with plasmids encoding Cas9 and two gRNAs (GCGTAAAAGTGCACGCGCCC and GAATCTAACCTTATCTGCCA). TMEM16F-KO cell lines were obtained by puromycin selection and manual picking of single colonies.

***WST-1 assay***

HEI-OC1 cells were seeded in 96-well plates with a density of 5×10^3^ cells/well. After 24 h, the cells were treated with various concentrations of ionomycin for 12 h or H_2_O_2_ for 6 h (500 μM H_2_O_2_ killed all cells at 12 h). The WST-1 cell proliferation and cytotoxicity assay kit (Beyotime, C0036) was used to evaluate cell viability. WST-1 reagent was added to the cell culture medium (10 μl/well) and incubated for 1 h at 37°C, and then the absorbance at 450 nm (A450) and 690 nm (A690) was measured using a microplate reader (Bio-Tek, Cytation 5). The value of A450-A690 was used to assess the cell viability.

***Flow cytometry***

HEI-OC1 cells were seeded in 6-well plates with a density of 1×10^5^ cells/well. After 24 h, the cells were treated with 5 μM ionomycin or 500 μM H_2_O_2_ (with moderate cytotoxicity) for 3.5 h (to mimic the 3.5-h noise exposure on mice). The cells were trypsinized and stained with Annexin V-FITC and PI in suspension using an Apoptosis Detection Kit (Beyotime, C1062L) following the manufacturer’s instructions. The cells were then analyzed in a flow cytometer (FACScan, BD Biosciences). Fluorescence of the cells were plotted and analyzed using the FlowJo 10.7.2 software (BD Biosciences).

***Western blot***

Mouse cochleae were homogenized and cells were lysed in RIPA buffer. Protein samples were electrophoresed in 10% polyacrylamide gel and transferred to PVDF membrane. Rabbit anti-mouse TMEM16F (Abcam ab234422, 1:1000) and GAPDH (Elabscience E-AB-40337, 1:1000) primary antibodies, and HRP-conjugated goat anti-rabbit IgG secondary antibody (Proteintech RGAR001, 1:2000) were used for immunoblot. Gel bands were visualized using ECL reagent (Epizyme SQ202L) and photographed in a gel document system.

***Statistics***

Data are presented as mean ± SEM (standard error of mean). Student’s *t* test was used in experiment with only two groups. One-way ANOVA and Tukey’s test were used to identify statistical differences among three groups. Two-way ANOVA and Sidak’s test were employed when the results were affected by two factors.
