# Supplementary material for: Deficiency of TMEM16F in hair cells prevents diabetes-related and noise-induced hearing loss
Source: Genes Dis. 2025 Jun 6;13(2):101708. doi: 10.1016/j.gendis.2025.101708 (PMC12648708; doi:10.1016/j.gendis.2025.101708)
Supplement: Multimedia component 3 [file mmc3.pdf]

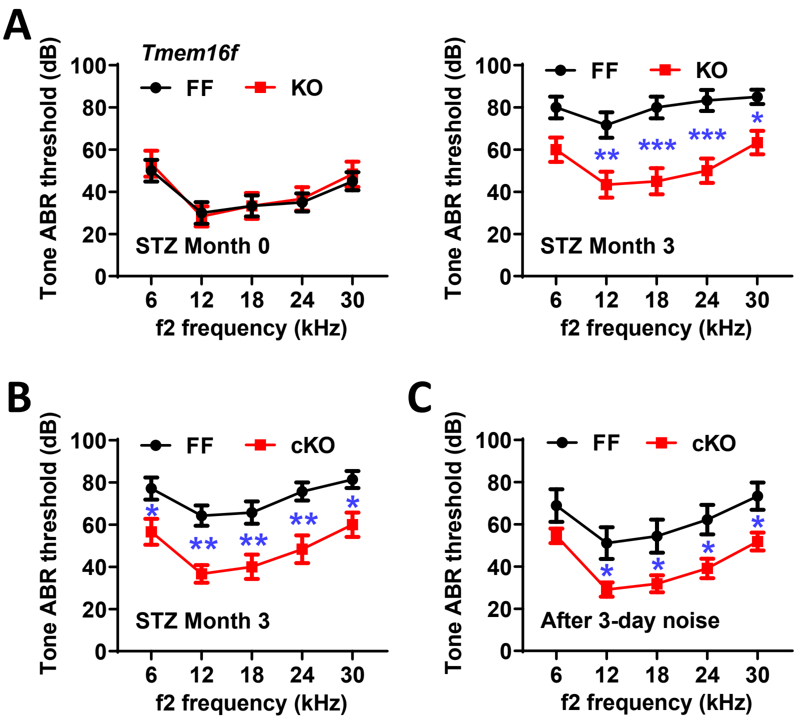

Figure S1. ABR thresholds evoked by pure-tone stimuli in mice at indicated timepoints. (A) *n*=6 mice in each group. (B) *n*=7 (FF) and 6 (cKO) mice. (C) *n*=9 (FF) and 11 (cKO) mice. **P*<0.05, ***P*<0.01 and ****P*<0.001, by 2-way ANOVA.

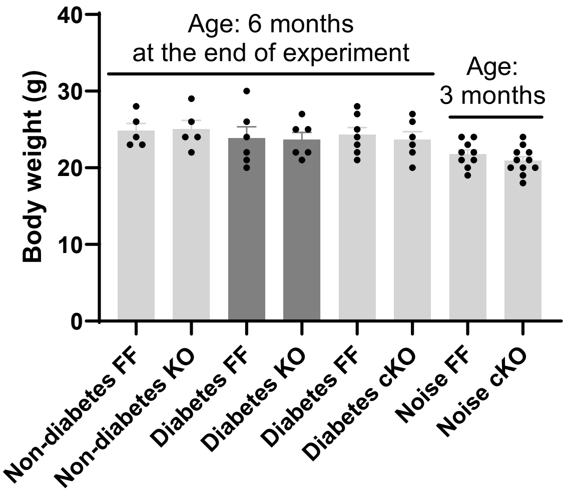

Figure S2. Body weights and ages of mice at the end of experiment.

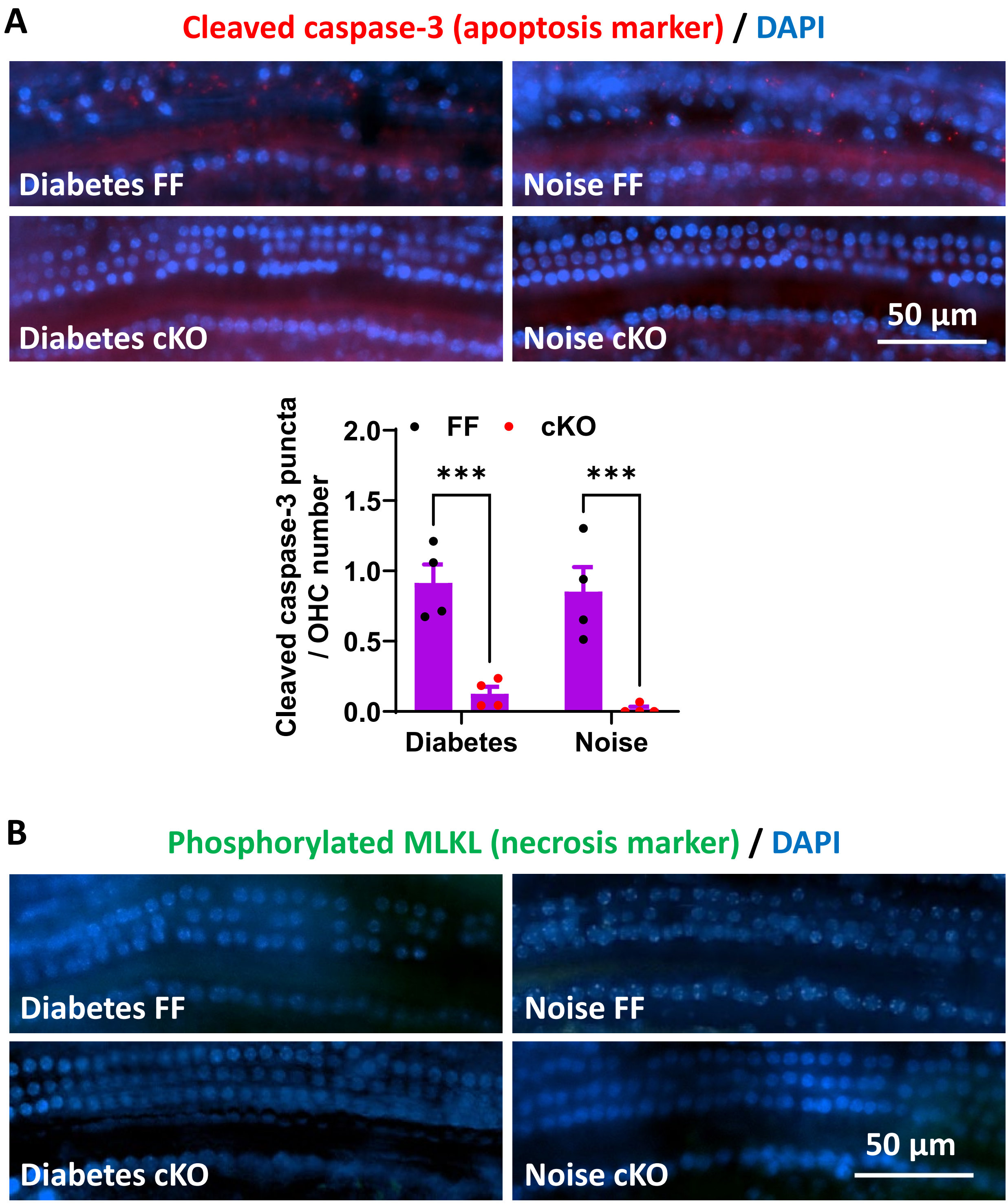

Figure S3. Apoptosis, but not necrosis of outer hair cells was found in FF mice, as indicated by positive immunofluorescence against cleaved caspase-3 and negative phosphorylated MLKL.

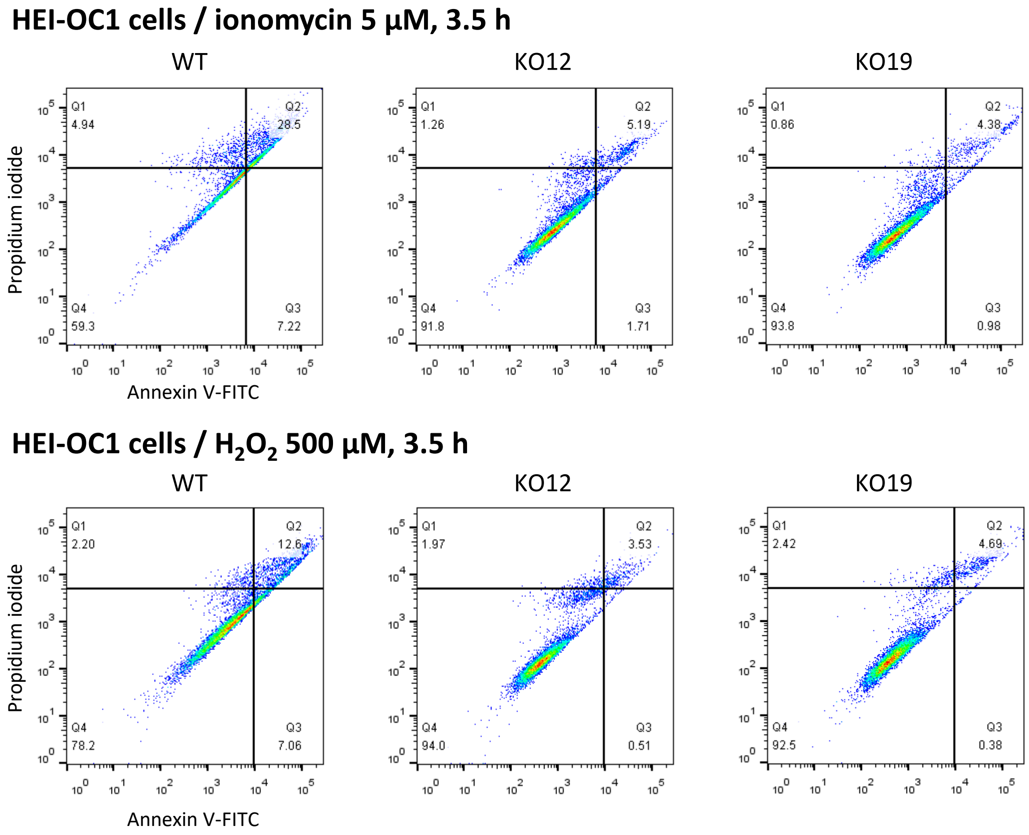

Figure S4. Flow cytometry dot plots for ionomycin and H_2O_2-treated WT and TMEM16F-KO HEI-OC1 cells stained with FITC-conjugated annexin V and propidium iodide.
